# Changing Malaria Prevalence on the Kenyan Coast since 1974: Climate, Drugs and Vector Control

**DOI:** 10.1371/journal.pone.0128792

**Published:** 2015-06-24

**Authors:** Robert W. Snow, Eliud Kibuchi, Stella W. Karuri, Gilbert Sang, Caroline W. Gitonga, Charles Mwandawiro, Philip Bejon, Abdisalan M. Noor

**Affiliations:** 1 Spatial Health Metrics Group, Kenya Medical Research Institute-Wellcome Trust Research Programme, Nairobi, Kenya; 2 Centre for Tropical Medicine & Global Health, Nuffield Department of Clinical Medicine, University of Oxford, Oxford, United Kingdom; 3 Eastern and Southern Africa Centre of International Parasite Control, Kenya Medical Research Institute, Nairobi, Kenya; 4 Centre for Geographic Medicine-Coast, KEMRI-Wellcome Trust programme, Kilifi, Kenya; Tulane University School of Public Health and Tropical Medicine, UNITED STATES

## Abstract

**Background:**

Progress toward reducing the malaria burden in Africa has been measured, or modeled, using datasets with relatively short time-windows. These restricted temporal analyses may miss the wider context of longer-term cycles of malaria risk and hence may lead to incorrect inferences regarding the impact of intervention.

**Methods:**

1147 age-corrected *Plasmodium falciparum* parasite prevalence (*Pf*PR_2-10_) surveys among rural communities along the Kenyan coast were assembled from 1974 to 2014. A Bayesian conditional autoregressive generalized linear mixed model was used to interpolate to 279 small areas for each of the 41 years since 1974. Best-fit polynomial splined curves of changing *Pf*PR_2-10_ were compared to a sequence of plausible explanatory variables related to rainfall, drug resistance and insecticide-treated bed net (ITN) use.

**Results:**

*P*. *falciparum* parasite prevalence initially rose from 1974 to 1987, dipped in 1991–92 but remained high until 1998. From 1998 onwards prevalence began to decline until 2011, then began to rise through to 2014. This major decline occurred before ITNs were widely distributed and variation in rainfall coincided with some, but not all, short-term transmission cycles. Emerging resistance to chloroquine and introduction of sulfadoxine/pyrimethamine provided plausible explanations for the rise and fall of malaria transmission along the Kenyan coast.

**Conclusions:**

Progress towards elimination might not be as predictable as we would like, where natural and extrinsic cycles of transmission confound evaluations of the effect of interventions. Deciding where a country lies on an elimination pathway requires careful empiric observation of the long-term epidemiology of malaria transmission.

## Introduction

Following the recent launch of a global effort to reduce the malaria burden in Africa [1–2], billions of USD overseas development assistance have been invested in scaling up vector control and efficacious drug delivery [3]. The changing political commitment, funding and intervention coverage have all been associated with modeled declines in malaria mortality [3–5]. This evidence has fueled a renewed expectation that global eradication can be achieved in the next few decades [6]. Malaria, however, is a vector borne disease, subject to intrinsic cycles of variation driven by climate, changes in human land use and the efficacy and coverage of interventions that target the parasite and vector. These factors vary in space, defining the diversity of malaria transmission across the African continent, and with time. One important lesson of the first Global Malaria Eradication effort, was that elimination cannot be achieved everywhere with the same interventions within the same time frame. It is therefore not surprising that not every country in Africa has witnessed a decline in malaria transmission since 2000, and that even within countries witnessing a decline, some areas have been resilient to change despite equivalent levels of vector control coverage within their national borders [7].

Without a clearer understanding of short-term and long-term cycles of malaria risk we might mistakenly attribute declining (or increasing) trends in malaria transmission to coincident interventions, and more importantly we will be unable to predict the future landscape of malaria risk necessary to plan for elimination. Here we analyze 40 years of data on *Plasmodium falciparum* prevalence along the Kenyan Coast, a geographical area subject to a single climate system and malaria control policy introduction. In the absence of a controlled experiment we examine long-term changes in malaria prevalence against a plausibility framework [8–9] that defines the temporal factors that might provide insight into long- versus short-term malaria cycles.

## Materials and Methods

### The Kenyan Coast

**Fig 1:**
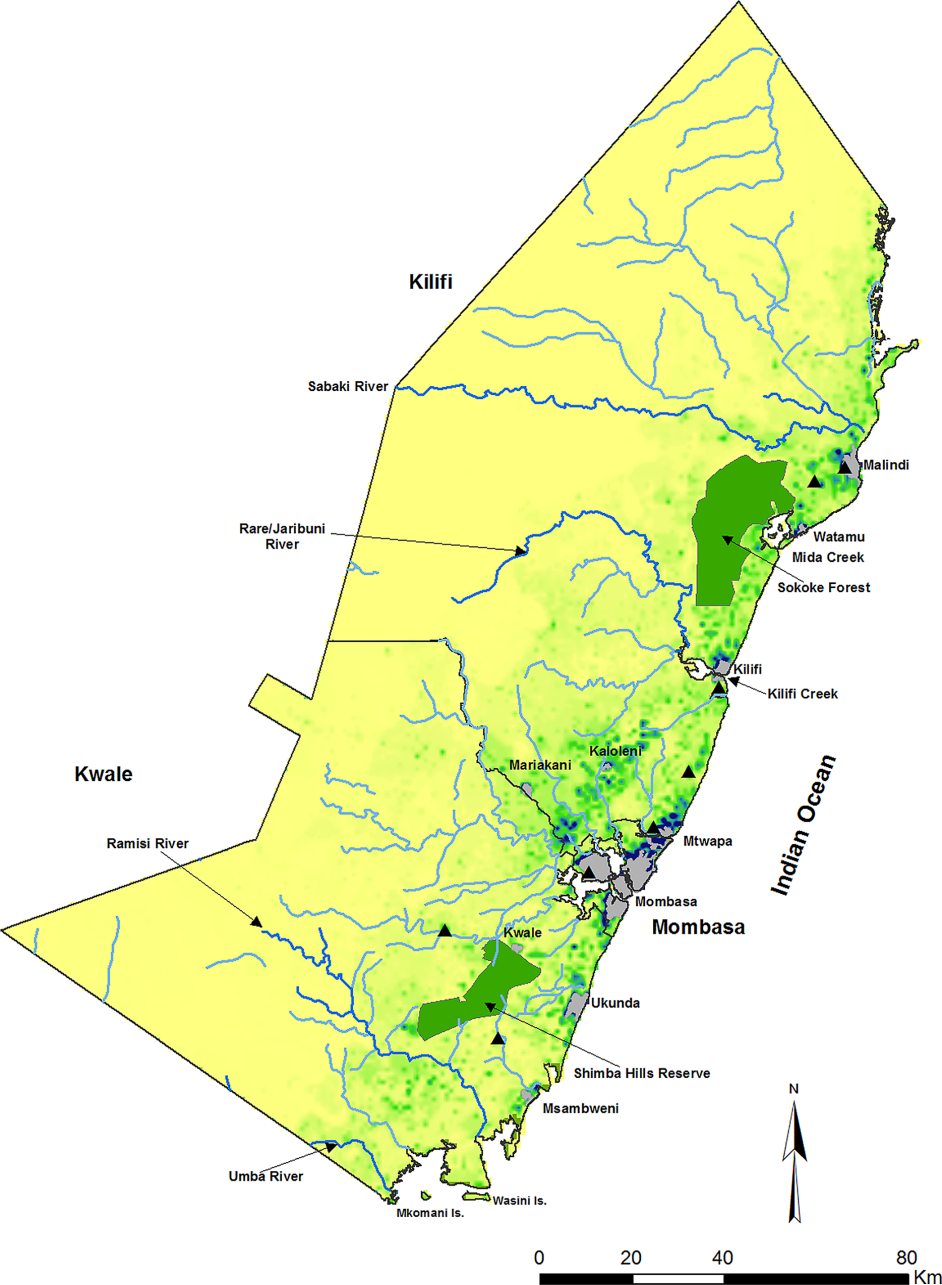
The Kenyan coast comprising of three counties (Kilifi, Mombasa and Kwale). Showing population density per 100 m^2^ (yellow 0 through dark blue 203 people per 100 m^2^) developed from high spatial resolution 1999 census data [13]; urban centres (Grey) defined by the national census bureau [14] where digitized boundaries undertaken using Google Earth, and used to exclude parasite prevalence data; the location of meteorological stations (Black triangles); major river systems (Blue).

Kilifi, Kwale and Mombasa Counties occupy 21,000 km^2^ of tropical moist deciduous forest, savanna and dry thorn bush, seasonal swamps and a number of plantations (sisal, coconut and cashew). The area is a flat plain with the highest altitude reaching 845 metres above sea level, coursed by three major river systems that feed into the Indian Ocean through networks of seasonal streams (Fig 1). 90% of the population reside within 50 km of the coastline, including ten major urban extents that cover Mombasa, Kilifi, Malindi, Mtwapa, Ukunda and Msambweni (Fig 1). The area is inhabited predominantly by the Mijikenda. The rural communities are largely subsistence farmers of maize, millet, cassava and beans. The major influence on the Kenyan coast's weather is the Inter-tropical Convergence Zone of the North East and South East Trade Winds. The hot northeast monsoon (*kaskazi*) from the Persian Gulf occurs from November to March/April and includes the ‘short rains’, variably between November and December. The moist monsoon blowing in from the southeast (*kusi*) occurs from April/May to October leading to the heaviest rain, referred to as the ‘long rains’ (March, April, May and June) [10]. The dominant malaria vector species groups are *Anopheles gambiae* and *An*. *funestus* [11–12]. The former predominates, with the sibling species *An*. *merus* occupying a narrow ecological niche close to the Indian Ocean coastline. Within the *An*. *gambiae* species group, *An*. *arabiensis* is more common in the northern parts of Kilifi County and *An*. *gambiae* s.s. (both M and S forms) is more common further south toward the Tanzanian border [12].

### Parasite prevalence survey data assembly

Information from parasite prevalence surveys undertaken since 1974 that formed part of routine surveillance or research enquiry among communities and school children in three coastal counties were assembled from national Ministry of Health archives, direct extraction from published reports or additional correspondence with research centres located on the Kenyan coast (S1 Text). Information from each survey included the date of examination, the age range of subjects included, the number examined, the number found positive for *P*. *falciparum* infection and the longitude and latitude of the survey location (S1 Text). Data were excluded if the survey could not be geo-coded (4), surveys were located in contemporary urban extents, to remove the long-term influence of urbanization on changing malaria risks (174), or where samples included less than 10 subjects (11).

### Spatial-temporal analysis

The three counties comprise of 279 sub-locations, the lowest administrative units, with a median area of 26 km^2^. Because of differences between surveys in the age ranges of sampled populations, the input *P*. *falciparum* survey data were corrected to a single standardized age range of 2–10 years (*Pf*PR_2-10_), using established catalytic conversion Muench models [15]. The rural parasite prevalence surveys were unevenly distributed in both time (month and year) and space (sub-location). To provide an analysis of temporal changes, adjusted for location of the survey, we used a Bayesian conditional autoregressive (CAR) generalized linear mixed (GLM) model with spatial and temporal effects to predict *Pf*PR_2-10_ for each of the 279 sub-locations for every year 1974–2014. The CAR-GLM model assumes that the number of children from 2 years to below 10 years of age that are positive in a sub-location and any time is a binomial random variable that is a function of the probability of infection and the number of children tested for malaria at each location and time. The logistic model incorporates the spatial autocorrelations as structured and unstructured spatial effects and time as a second order autoregressive effects within each of the 279 polygons. The structured and unstructured spatial effects were assigned a Markov random field prior and independent and identically distributed Gaussian respectively. The temporal random effect was assumed to be random walk model of order 2. Bayesian inference was achieved using integrated nested Laplace approximation (INLA) in R [16–17]. Model posterior output included the median, the 25^th^ and 75^th^ percentiles of *Pf*PR_2-10_. Full model specifications are provided in the S1 Text and model outputs provided in S1 Data for each sub location (S2 Data). A Generalized Additive Model [18] was used to smooth the 279 annual posterior median predictions of *Pf*PR_2-10_. The GAM model assumed a Gaussian distribution for the median, the 25^th^ and 75^th^ percentiles of the predicted *Pf*PR_2-10_ and applied a segmented cubic polynomial smoothing spline.

### Assembly of plausibility factors

Monthly rainfall data, January 1970 to December 2014 (45 years), were assembled from eight range gauges situated across the densely populated areas shown in Fig 1. Complete data was available for 4130 (96%) of the 4320 possible months. Monthly means across each of the meteorological sites were computed to provide long-term percentage anomalies for each year and for each March-June long rains, linked to changes in sea surface temperatures in the Indian Ocean that define climate patterns along the East African coast [19].

The annual quantities of nets, re-treatments and long-lasting net distributions were assembled from month-commodity-location specific databases of social marketing agencies and the Ministry of Health between 1999 and 2014. We have assumed that all nets in circulation since May 2005 have been Long-Lasting Treated Net (LLIN) [20]. Nets before 2005 would have required re-treatment every six months and we have presumed effectiveness only for one 12 month cycle for each net distributed. For LLIN we have assumed, in line with expert opinion, that 92% of LLINs would remain optimally "efficacious", retention of pyrethroid concentration and durability of netting, during the first year of use; 80% during the second year of use; 50% during the third year of use; and ineffective in year 4 [21]. Data were assembled to show cumulative monthly distributions and availability of "effective" nets per capita population derived from population census growth models 1989-1999-2009 [14, 22] (S3 Data). Data on reported use of an ITN the preceding night by members of all ages within rural clusters of sampled households were identified from seven national surveys, in 2003, 2005, 2007, 2008, 2009, 2010 [23–28] (S3 Data). For 2014, we have used unpublished data on the reported use of an LLIN last night recorded during surveys of 11618 school children attending 117 rural schools along the Kenyan coast [Mwandawiro & Snow, unpublished data].

Using published and unpublished data from various sites along the Kenyan coast we have, where possible, represented the speed of emerging chloroquine (CQ) and sulphadoxine-pyrimethamine (SP) failure as the proportions of parasitaemic children receiving standard therapeutic doses unable to clear infections, or observed recrudescence, by day 7. Data used to construct the plausibility framework is provided in S3 Data.

### Ethics

Here we have used previously published and unpublished data. For each survey various ethical approvals were sought from national institutional review boards. Where possible these have been listed in the Supporting Information accompanying the search procedures for identifying each survey report (S1 Text). In no instance were individual patient level data used, only aggregates per village or school cluster.

## Results

### Cycles of changing parasite prevalence: 1974–2014

**Fig 2:**
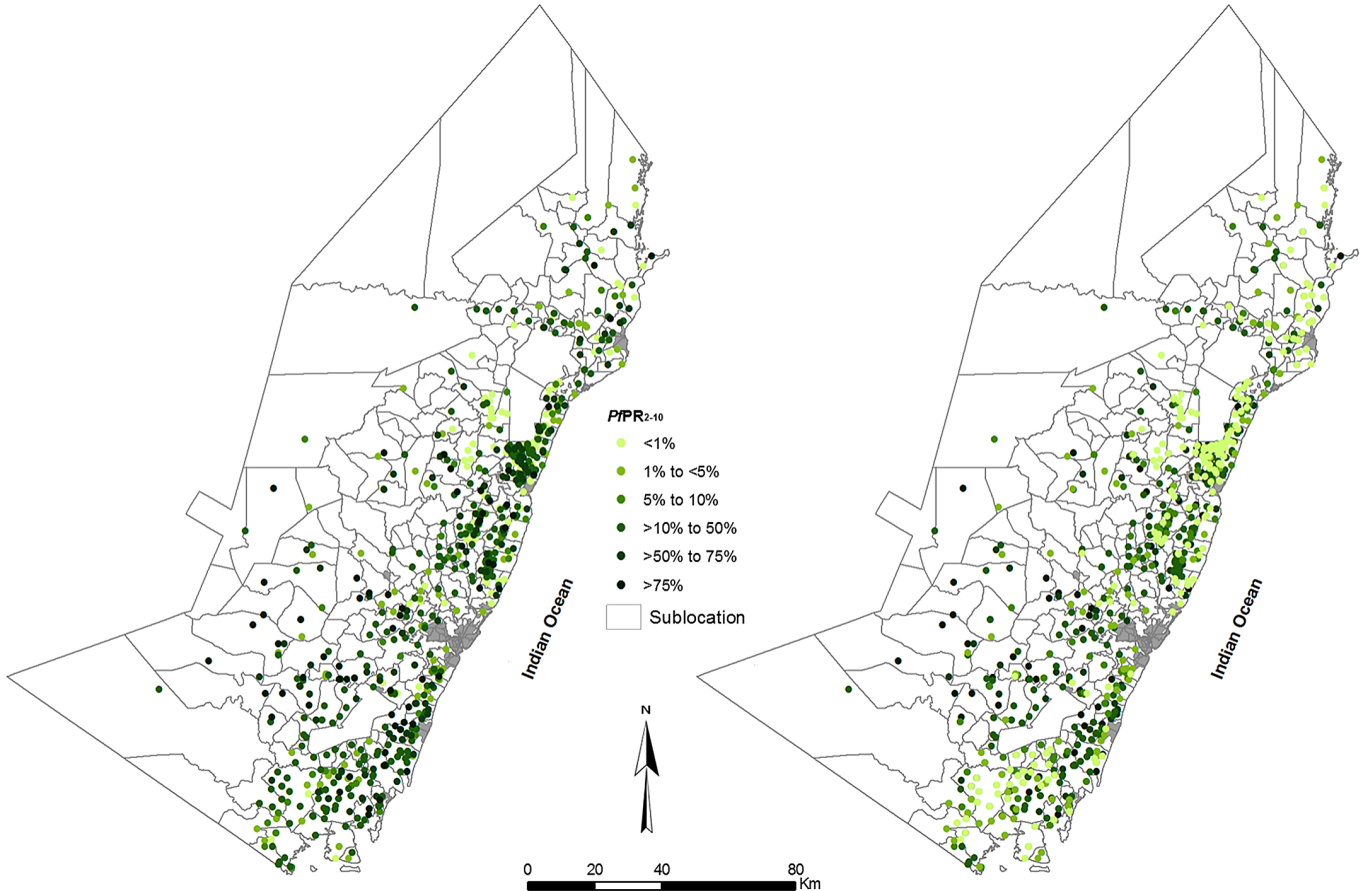
Location of age-corrected parasite prevalence (*Pf*PR_2-10_) with the highest recorded estimate of prevalence on top (Left hand panel) and lowest *Pf*PR_2-10_ estimate on top (Right hand panel) to distinguish prevalence at similar locations with time. Data displayed against 279 fifth level census administrative units used to make monthly median malaria predictions (see Methods).

**Fig 3:**
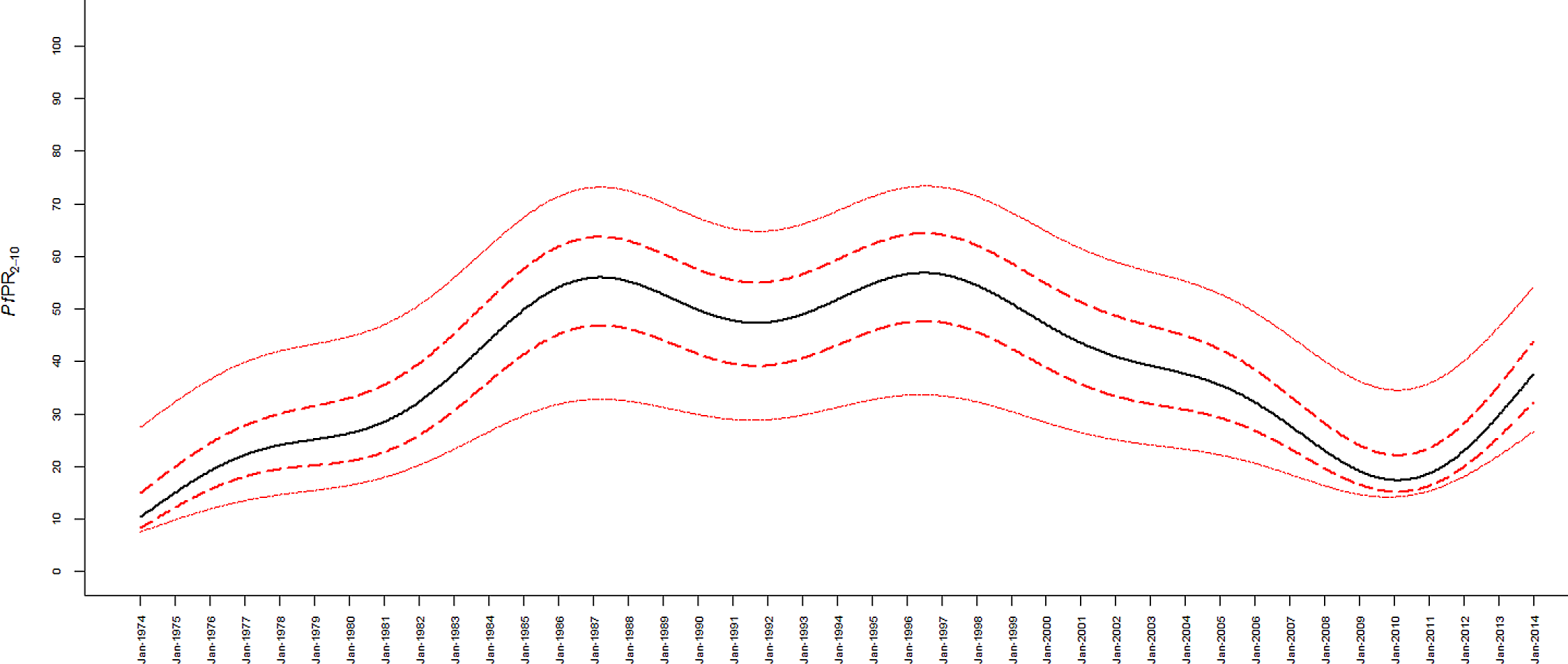
Median small area annual estimates of *Pf*PR_2-10_ for the 279 sub-locations fitted using a Generalized Additive Regression model between January 1974 and December 2014 (black line). The 25% and 75% inter-quartile range and the 2.5% and 97.5% credible intervals are shown as solid and dashed red lines respectively.

We have assembled information on the prevalence of *P*. *falciparum* infection from 1144 surveys undertaken among rural communities along the Kenyan coast between 1974 and 2014 (Fig 2), making it one of the richest long-term time-series data on malaria prevalence within a constrained area anywhere in Africa. Using a Bayesian model-based geostatistical approach applied to each time and geo-located prevalence survey within 279 administrative polygons we show that parasite transmission intensity initially rose in two steps, gradually from 1974 to 1980 and more sharply from 1982 to 1987 (Fig 3). *P*. *falciparum* parasite prevalence remained high for at least another decade, with a dip 1991–1992. From 1998 prevalence began a decline through to 2010, corresponding to previous reports of declining paediatic malaria admissions in Kilifi [29–30], and declining admissions at hospitals in Malindi and Msambweni [30] and declining infection prevalence among pregnant women in the southern part of the coast [31]. The decline ends in 2011 and begins to rise through to 2014 (Fig 3).

### A plausibility framework

**Fig 4:**
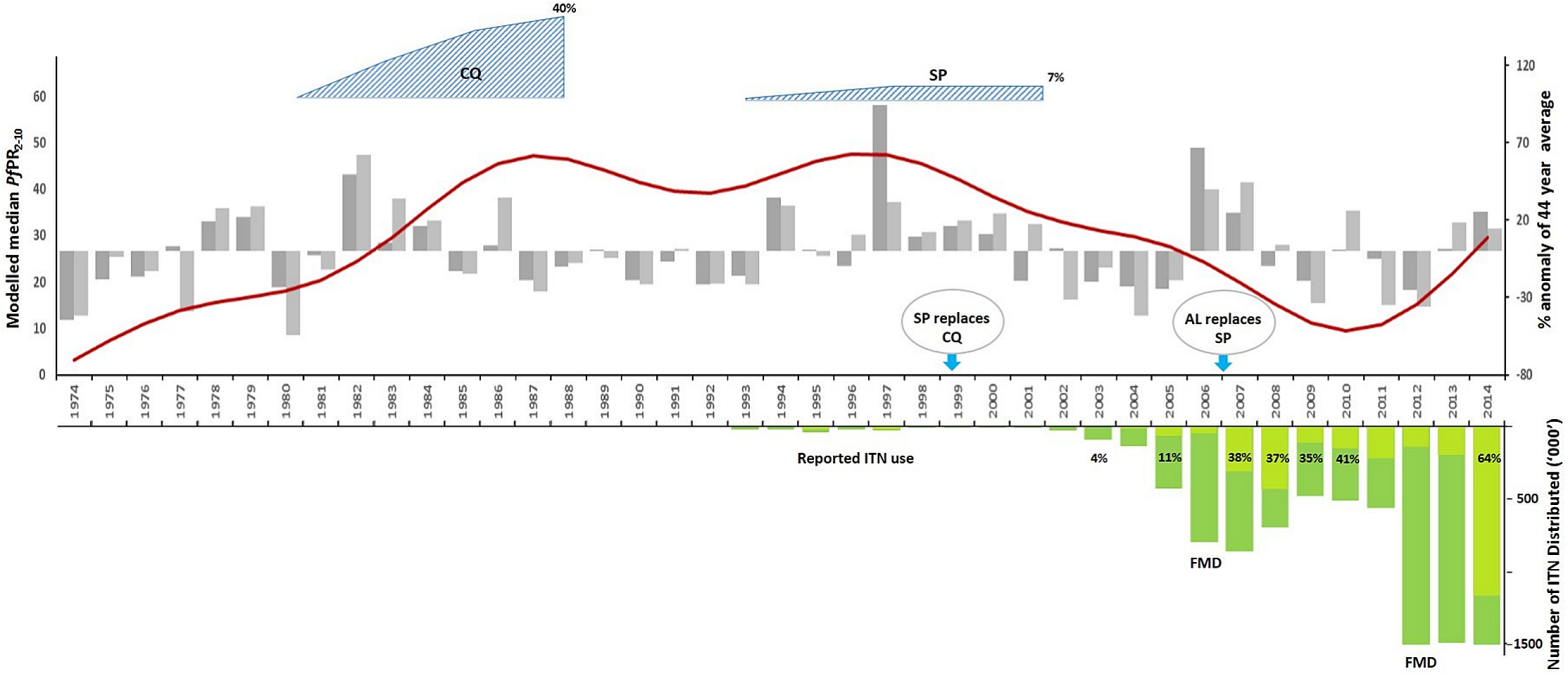
Plausibility framework. Extracted monthly GAM fitted median *Pf*PR_**2-10**_ (red line) shown in relation to annual and long rains (March-June) percentage anomalies in precipitation (dark and light grey bars respectively); cumulative "effective" mid-year ITN distribution data (dark green likely efficacious, light green <50% efficacious, see methods); estimated day 7 anti-malarial drug failures to clear parasitaemia based on information provided in text (blue triangles); and malaria policy milestones for standard treatment guidelines and mass ITN distribution dates (FMD), including reported ITN use among all age groups (S3 Data).

Fig 4 considers the combination of factors which might explain the changes witnessed in parasite prevalence since 1974.

#### Rainfall

The period 1974 to 1981 was characterized overall by lower than average long-term annual and long-rains precipitation, while malaria prevalence rose from 1974 to 1978. Excess rainfall witnessed in 1982, followed by years of higher than average rains might have contributed to the increasing malaria prevalence curve through to 1987. The high malaria prevalence through to 1993 was however maintained during periods of drought. Abnormally high rainfall occurred in 1994, and the El Niño excessive rains in 1997, both correspond with peaks in parasite prevalence within the decade-long plateau. The subsequent downward trajectory of parasite prevalence through to 2011 occurred at a time when rainfall was above normal, but continued its decline from 2002–2006 at a time marked by drought. Heavy rainfall in 2006 and 2007 did not seem to be associated with any discontinuity in the continued decline in parasite prevalence through to 2010, however the drought years of 2008–2010, typical of East Africa at this time [19] may have contributed to the lower malaria prevalence in this interval. Parasite prevalence began to rise from 2011 during the continued drought and before the above average rainfall in 2014.

#### Vector control

The first use of insecticide treated nets (ITN) on the Kenyan coast was between 1993–1995 as part of randomized clinical trials in an area north of Kilifi Creek [32] and in the southern parts of Kwale county [33]. Between 1995 and 2003, social marketing of ITN and re-treatment of nets achieved very low coverage [20, 34]. From October 2004, ITNs were offered through maternal and child welfare clinics free of charge. In October 2006, the first mass, door-to-door campaign to deliver free LLIN as part of a vaccine catch-up strategy was launched [20]. Routine delivery of ITN continued in subsequent years until a further mass campaign in March 2012, followed by routine delivery through to December 2014. LLIN distribution only achieved moderate coverage late in 2006 and maintained these levels until March 2012. Reported use of an LLIN by members of the community, 2007–2010, was only 35%–40% (Fig 4). This might have contributed to an already declining prevalence witnessed during this period, however it is implausible that the declines from 1997 through to 2005 could have been associated with ITN distribution, since in 2003 only 3% of rural household members reported using an ITN.

The mass campaign in March 2012 delivered over 1.5 million LLIN to communities along the coast. This was the largest single delivery of LLIN, there are no community level data on coverage, however, 68% of school children reported using a treated net the night before surveys in 2014. By mid-2014 (28–34 months since delivery), many of these LLINs would have been on the margins of optimal effectiveness and not adequately replaced though routine distributions. Plans to replace LLINs through repeat mass campaigns in the Coastal region in 2014 were delayed and are now planned for 2015. Bioassays of vector susceptibility to pyrethroids in 2012 showed greater than 95% mortality after 24 hours [Mbogo CNM, unpublished data]. We do not have any recent information on behavioural adaption of local dominant vectors to ITNs on the Kenyan Coast. Immediately following the introduction of ITN in Kilifi in 1994, earlier biting outdoors was recorded [35]. Elsewhere in Kenya the wide-scale use of ITN has been associated with changing *An*. *gambiae* sibling species compositions and feeding behaviours [36]. Nevertheless, malaria prevalence continued to rise from 2011 to 2014, reaching levels higher than those witnessed in the 1970s when vector control was largely absent.

#### Drug use and efficacy

The use of CQ and pyrimethamine as prophylaxis or mass-drug administration were reported at settlement schemes at Sabakai-Malindi in Kilifi County and Shimba hills in Kwale County as well as part of fortnightly school "treatment parades" during the early 1970s. Retail sector use of CQ for self-medication had been common for many years [37–38]. Between 1993 and 1995, 14% of aparasitaemic, afebrile and 44% of febrile children from the community around Kilifi had detectable levels of CQ in their blood [39]. The first case of CQ resistance in Kenya was reported in 1978 in a non-immune tourist [40]. *In vitro* CQ resistance was detected in semi-immune children along the Kenyan coast in 1982 and first reports of *in vivo* resistance in 1983 [41]. Resistance among the local parasite populations expanded rapidly, with sequential data suggesting that by 1987 more than 40% of infected children failed to clear infections within 7 days with standard 25mg/kg base three day treatments [42–44] (Fig 4).

In 1999, CQ was replaced as first line recommended treatment with SP [45], although in practice SP was being used before this date [38] and CQ was still used by some for periods after this date [23,46]. Parasites were fully sensitive to SP in 1987, with all infections being cleared within 7 days and patients remaining uninfected through day 14 [47]. There are fewer longitudinal data on SP resistance from the coast, however *in vitro* and *in vivo* SP resistance was reported in 1993 [39,48–49], early treatment failures were 7% in 1997 [50] and failure to clear infections by day 14, or early treatment failure, remained at 4–7% by 2000–2001[51–52] (Fig 4). Lower rates of resistance and SP treatment failure were described on the Coast compared to other areas of Kenya by 2001 [52]. The national treatment policy was changed in 2004 recommending the use of Artemether-Lumefanthrine (AL) as first line malaria therapy, however drug supply, revised guidelines and training were not implemented until the end of 2006 [53] and SP was still being used for treatment up to the beginning of 2007 [54].

It seems plausible that the rapid emergence of resistance to CQ from the early 1980s contributed to the rising parasite prevalence through to the 1990s. Conversely, SP resistance may not have spread as quickly, and while failing to adequately clear infections its continued use through to 2007 might have nevertheless had a prophylactic effect that paradoxically contributed to the declines in malaria prevalence during the 2000s. The triple mutant *pfdhfr*, encoding dihydrofolate reductase that confers biological resistance to SP (51I/59R/108N haplotype) was only 37% in 1999 in Kilifi [55]. The *pfdhfr* quadruple mutant, that renders SP ineffective, has not been described on the Kenyan Coast [55–56]. SP has a long half-life providing periods of prophylaxis following single dose administration. On the one hand this long half-life is a threat to drug resistance since parasites are exposed to low drug concentrations, but on the other hand may have a benefit in a prophylactic effect which reduces parasites while they remain sensitive [57]. Before SP was replaced with AL, it had not reached complete failure and was widely available in the retail and private sectors [46,54]. By 2010, SP use for fevers was non-existent on the coast, although only 24% of fevers in young children were treated with AL [28].

## Discussion

Malaria parasite prevalence data assembled over 40 years along the Kenyan coast show cycles of change. These long-term cycles provide an opportunity to examine the temporal effects of rainfall, vector control and anti-malarial drug use. It is perhaps most notable that *P*. *falciparum* prevalence was lowest at a time when any specific efforts to prevent infection through vector control were largely absent but malaria control relied solely on the wide-spread use of CQ or pyrimethamine for treatment, or occasional mass administration, during the early 1970s. The 1970s and early 1980s were characterized by lower than average annual and long-rains (with the exception of 1982), but rapidly expanding CQ failure rates. We presume that the latter explains the escalating parasite prevalence that was observed. For over a decade malaria prevalence remained high, with peaks and troughs that correspond with variations in rainfall patterns. A decline was observed between 1998 and 2003 throughout which ITN coverage was extremely poor. Rainfall during this period was average, or above-average, with the exception of a period between 2003 and 2005. This period of decline, however, had one distinguishing feature, SP had replaced CQ for presumptive fever management and continued to be widely used up until 2007.

Despite the rapidly changing landscape of ITN use following the October 2006 mass campaigns and sustained free distribution through routine clinics, the reported use of ITN by all-age groups among rural communities on the Kenyan coast only reached 43% by 2010. This raises two important aspects of plausible attribution: first, the significant declines in malaria prevalence started well before significant changes in ITN access; second, while individual clinical protection is likely to have been afforded for those using an ITN during its effective life-span, the mass effects on parasite transmission would have been minimal before 2012. Theory suggests that >80% effective coverage (100% efficacious ITN, used every night by every household occupant) is required to halve infection prevalence in areas where the natural prevalence is *circa* 40% [58–59]. Equally, the rising parasite prevalence from 2011 through to 2014 occurred during a period of significant increases in the delivery of LLIN in March 2012 and higher personal use. However, it is possible that the LLINs distributed in 2012 were no longer effective by 2014, when higher than average rainfall was reported. Hence although malaria transmission would no doubt have been higher still without the mass LLIN distributions, ITN distributions do not provide an adequate explanation for the trends in malaria prevalence since 1998. These observations correspond with those reported from a single village in Senegal 1990–2012, in some ways a more readily interpreted set of observations, where prevalence began to decline before large-scale increases in ITN but at a time when CQ was replaced by a combination of amodiaquine+SP for treatment [60]. We re-emphasize that we are not suggesting that LLIN, when used under optimal conditions do not provide important, immediate clinical protection against malaria. The evidence for protective efficacy is overwhelming from the Kenyan Coast [32] and elsewhere [61]. Rather we are suggesting that under operational conditions the impact of LLINs may be relatively minor when coverage is lower than trial conditions and when set against more powerful secular trends.

The ubiquitous use of long half-life drugs such as CQ or SP, and the acquisition of resistance to these drugs, cannot be ignored as plausible explanations for the low infection rates at the beginning of the time series or the period of declining malaria post-1998. The use of drugs as adjuncts to transmission control has a long history in Africa [62–63]. While the intention of CQ and SP use was largely for treatment only, both drugs were readily available and used in great quantities by the population who had ready access to these cheap drugs at clinics and over-the-counter through the retail sector. After 2007, SP was rarely used for fever treatment, although still used to presumptively treat pregnant women. More importantly, since September 2010, revised standard treatment guidelines have restricted AL to the management of parasite confirmed febrile illnesses presenting to the formal health sector [64], a treatment source used by only 35% of fevers [54]. By 2012, this policy was effectively implemented along the Kenyan Coast [64]. Although this is a sensible clinical policy for the use of expensive treatments and reduces the drug pressure on the parasite population, AL will not have the same level of contact with circulating symptomatic and asymptomatic parasites as either CQ or SP did and therefore may be associated with increasing transmission.

Fears of resistance have always mitigated against the wide-scale use of anti-malarial drugs. However, if combined with at least 90% effective coverage of vector control and if we can avoid mass treatment with the same anti-malarials that we rely on to treat clinical illness, then mass treatment might sustain declines in malaria transmission that began in the late 1990s and reverse the resurgence in prevalence seen in recent years.

The intensity of malaria parasite transmission along the Kenyan coast is a fragile and complex system. The rise, fall and rise may well form part of much longer transmission cycles not captured within the present 40 year series. The first surveys undertaken on the coast were in Digo County (now Kwale) in 1932 [65], around Mombasa Island in 1937 [66], at Durama (Kwale County) in 1952 [67], and by the Division of Insect Borne Diseases of the Ministry of Health in 1958 around Malindi [68]. Among 24 communities sampled between 1932 and 1958, mean *P*. *falciparum* infection prevalence was 67% (Range 26%-96%) and 17 of the sampled communities had infection prevalence above 50%. As such the prevalence during the 1970s might have been part of a trough, and the peak "epidemic" prevalence recorded during the 1990s a more, historical "natural" level of parasite exposure.

Long-term data within specific areas of Africa allow us to unpack the complexity of transmission cycles with time and provide a framework to interpret change in relation to intervention and climate. There are, however, important caveats. Plausibility analyses are constrained by the amount and timing of available data, which are easier to assemble for rainfall, but considerably harder to compile for periodic surveys of drug sensitivity, drug use, insecticide susceptibility or the true reported use and effectiveness of personal protection measures. Here we have presented what evidence exists to provide a series of temporal plausibility explanations for the changing patterns of parasite prevalence.

We highlight two important considerations for the proposed global malaria eradication strategy, when applied to Africa; first elimination end points might not be as predictable as we would like, with natural and extrinsic cycles of transmission confounding models that make too many assumptions based on very little empirical evidence; and second it will be unacceptable in future to rely on a plausibility analysis of imperfect data to decide where on an elimination pathway a region within a country currently lies and has transitioned from. We must purposively collect longitudinal data on transmission, the precise effective coverage of control and contextual data on behavioural (drug access/use or vector feeding patterns) and efficacy (biological responses of vectors to insecticides and parasites to drugs) if we are to make more tangible sense of malaria control and elimination efforts over the next two decades.

## Supporting Information

S1 Text Background to data assembly and models. (DOCX)

S1 Data INLA Predictions. (CSV)

S2 Data sublocations_shp (ZIP)

S3 Data Plausibility timeline data. (XLSX)
