# Supplementary material for: Changing Malaria Prevalence on the Kenyan Coast since 1974: Climate, Drugs and Vector Control
Source: PLoS One. 2015 Jun 24;10(6):e0128792. doi: 10.1371/journal.pone.0128792 (PMC4479373; doi:10.1371/journal.pone.0128792)
Supplement: S1 Text — (DOCX) [file pone.0128792.s001.docx]

**Supplementary Information 1**

**Survey data assembly**

The proportion of individuals infected with *Plasmodium falciparum* has been a surveillance metric for malaria in Kenya since the establishment of the Ministry of Health's (MoH) Division of Insect/Vector Borne diseases (DVBD) in the 1940s. These surveys formed part of routine malaria reconnaissance and longitudinal malaria control impact assessments. Since 1975 most DVBD surveys were among school children until government funds were restricted for the Division in the late 1980s. All monthly and annual surveillance reports from Ministry of Health field stations along the coast held at archives in Nairobi, Kwale and Mombasa were identified. Nationwide, school-based malariometric surveys were resurrected as a surveillance strategy in 2007 [1-2].

Survey data on malaria infection prevalence among community residents investigated as part of immunological, epidemiological or intervention studies of malaria, or indirectly as part of other infectious disease enquires, were assembled from published and unpublished data from areas within a demographic surveillance site located south of Mida Creek and immediately south of Kilifi creek in Kilifi County as part of the research programme of the KEMRI-Wellcome Trust [3] and epidemiological studies undertaken by the KEMRI-Case Western University collaboration [4] around Msambweni (Kwale County). In 2007 and 2010, national household cluster samples were surveyed as part of a malaria indicator survey [5-6].

For each survey location, the longitude and latitude was established from Global Positioning System recordings or high resolution digital atlases. Data derived from randomized controlled intervention trials, were only included for pre-intervention and subsequent follow-up cross-sectional surveys among control populations. The minimum required data fields for each record were the start and end dates of the survey (month and year), information about blood examination (number of individuals tested, number positive for *Plasmodium* infections by species), the methods used to detect infection (microscopy, Rapid Diagnostic Tests (RDTs), Polymerase Chain Reaction (PCR) or combinations) and the lowest and highest age in the surveyed population.

**Ethical approval**

There is no record of national ethical approval for early surveys of infection prevalence, as these formed part of routine surveillance. Data derived from research studies undertaken since 1989 include the following protocols and national ethical approval numbers: Case control studies to identify the role of host, parasite and environmental factors in determining the severity of clinical malaria (KEMRI SCC/ERC 135); A trial of insecticide treated bed nets in the reduction of childhood mortality and morbidity on the Kenyan Coast (KEMRI SSC/ERC 244); The natural history of acquired immunity to malaria with particular reference to responses to *P. falciparum* variant antigens expressed at the infected red cell surface (KEMRI SSC/ERC 359 & 485); Evaluation of three MOH district demonstration programmes for training informal antimalarial drug retailers (Kwale, Busia and Makueni) (KEMRI SCC/ERC 746); Integrated studies of the development of natural immunity to malaria in children in Kilifi district (KEMRI SCC/ERC 1131); An effectiveness study in Kilifi District of 7-valent pneumococcal conjugate vaccine administered through the routine childhood immunization programme (KEMRI SCC/ERC 1433); Extended follow up of a Phase IIb vaccine trial with RTSS in Kilifi District, Kenya (KEMRI SCC/ERC 1512); Adaptive, integrated malaria vector management in urban and peri-urban Malindi (KEMRI SCC/ERC 1725 & 2675); Case Western Reserve University, Cleveland, OH, USA and KEMRI collaborative studies 1996-2014 (KEMRI SSC/ERC 1040); School surveys since 2014: Kenya Medical Research Institute and National Ethics Review Committee (KEMRI SSC/ERC 1407, 1596 & 2801); National Malaria Indicator Surveys in 2007 and 2010 received ethical approval from the Kenyatta National Hospital/University of Nairobi Scientific and Ethical Committee. In no instance were individual level records used for the present study.

**Acknowledgements**

The following scientists have provided invaluable assistance in providing site specific data from published and unpublished investigations along the Kenyan coast: Timothy Abuya, Philip Bejon, Donal Bisanzio, Simon Brooker, Mark Divall, Lia Smith Florey, Carol Gitonga, Laura Hammitt, Jimmy Kihara, Alice Kamau, Charles King, Chris King, Astrid Knoblauch, Bret Lowe, Vicki Marsh, Margaret McKinnon, Charles Mbogo, Moses Mosobo, Charles Mwandawiro, Joseph Mwangangi, Tabitha Mwangi, Chris Nevill, Vikky Nyaga, George Nyangweso, Edna Ogada, Milka Owuor, Antony Scott, Bob Snow and Juliana Wambua. In addition, Eric Muchiri, Elizabeth Juma and Rebecca Kiptui facilitated access to Ministry of Health archives or provided direct access to national household sample survey data; and Victor Alegana, Viola Kurui, Betsy Makena, Caroline Kabaria and Lydiah Mwangi provided help locating archive materials and geo-locating survey sites.

**Summary of data**

Four survey locations in Kwale County, sampled in 1992, could not be geo-located from the information provided in the original DVDB reports. Eleven surveys sampled less than 10 individuals between 1989 and 1990 in Kilifi County and these have been excluded from the analysis. 174 surveys were sampled in areas that we have classified as urban in 2009, some may have been sampled in what were rural peripheries prior to the 2009 urban extents, however, we aimed to remove the influence of urbanization on infection prevalence from our analyses and have restricted analysis to only surveys undertaken in areas classified as rural since 1974. The remaining 1141 surveys undertaken covered 105,193 individuals living in 823 unique locations. 204 communities or schools were seen at least twice. 667 (59%) of surveys were among school children at 334 schools between 1975 and 2014. 80 (7%) surveys at the community level were undertaken as part of routine investigations by DVBD between 1974 and 1994. 283 (25%) time-space survey locations were sampled between 1989 and 2013 as part of various research surveys of the KEMRI-Wellcome Trust, Kilifi County. 28 (2%) survey clusters were included in the national Malaria Indicator Surveys of 2007 (12) and 2010 (16). Unpublished data from *ad hoc* surveys undertaken along the coast by research scientists and private organizations provided information on 53 (5%) surveys locations. Eight peer reviewed publications provided an additional 30 (3%) survey locations, conference abstracts (2), local doctoral theses (2). Across all surveys, 753 (66%) were among sampled populations of 50 or more, 439 (38%) among sampled populations of 100 or more. The median sample size across all 1141 surveys was 67 (IQR: 44,100).

277 (24%) of the surveys used RDTs, between 2003 and 2014: Paracheck (Device & dipstick) (158); OptiMal (63); Rapid Uni-Gold (41); CareStart Malaria Pf/Pv Combo (12); and ICT (5). Of those where RDTs were used, 90 (40%) were additionally slide confirmed (all positives re-checked and 10% sample of negatives). Standardizing between diagnostic methods is not possible, there are no accompanying metrics with each survey description to judge the quality of microscopy (e.g. quality of staining, slide storage, how many high power fields examined, and magnification of microscopy) nor any reliable algorithm to standardize between different RDTs or between RDTs and slide readings. Previous examinations of the reliability of RDTs versus slide reading during surveys of Kenyan school children showed a close correlation between methods, however assuming microscopy as the gold standard was equally challenging given discordance rates between slide readers [7]. Under controlled conditions of quality assured microscopy the WHO-FIND team have suggested that most RDTs have equivalent sensitivity and specificity to microscopy slide reading [8].

Survey data are summarized in Table below.

SI Table 1: Number of surveys [unique sites]

|  | **Kilifi**  **County** | **Mombasa County** | **Kwale**  **County** | **Total** |
| --- | --- | --- | --- | --- |
| **1974-1989** | 117 [77] | 2 [2] | 40 [32] | 159 [111] |
| **1990-1999** | 104 [98] | 2 [2] | 30 [27] | 136 [127] |
| **2000-2004** | 42 [21] | 2 [2] | 58 [56] | 102 [79] |
| **2005-2009** | 133 [133] | 2 [2] | 40 [39] | 175 [174] |
| **2010-2014** | 206 [175] | 11 [6] | 352 [151] | 569 [332] |
| **Total** | 602 [504] | 19 [14] | 520 [305] | 1141 [823] |

**Small area estimation (SAE) using Generalized Linear Mixed Models (GLMM)**

SAE methods handle the problem of making reliable estimates of a variable of interest at areal units by accounting for the different random effect structures (structured spatial, unstructured spatial and temporal) in the data, smooth the variance due to sampling (“smoothing” of extreme rates a result of small local sample size) and compute estimates for areas without data by using data from sampled areas in the neighbourhood [9-12]. These methods were used to provide estimates of *Pf*PR_2-10_ at the small area (sub-location) using a hierarchical generalized linear mixed model (GLMM) within a Bayesian framework using Integrated Nested Laplace Approximation (INLA) [13] for inference. Posterior estimates of the median, inter-quartile range and the 2.5% and 97.5% percentiles of the *Pf*PR_2-10_ were computed for each sub-location for each year from 1974-2014. Covariates were not used in the computation of the small area estimates given the distribution of the data and to ensure that the predicted cycles are unaffected by problems of over-fitting. Furthermore, this allowed for the independent visual comparison of the predicted cycles in malaria transmission and those of the climatic, environmental, intervention and policy milestones.

In brief, the prevalence of *P. falciparum* calculated from of the malaria positive cases and the total examined individuals was modelled using a hierarchical GLMM accounting for spatial and temporal dependencies. The data were treated as binomial and modelled via the logit link function. Considering $N_{it}$to be the number of individuals tested for malaria, and $Y_{it}$ the number who tested positive at site $s_{i},i=1,...,n$ and at year $t=1,...,41$ in years. Then $Y_{it}$ was assumed to be distributed binomially $Y_{it}\sim Bin(N_{it},p_{it})$ with parameters $p_{it}$ measuring the proportion of positive cases at location $s_{i}$ and year$t$. A logistic regression model was used to relate the proportion of positive cases with the spatial and temporal random effects via the equation $\text{logit}(p_{it})=u_{it}+v_{t}$ where $u_{it}$the spatial random effects is, $v_{t}$is the temporal random effects and no fixed effects were considered in the formulation. The Besag model was assumed for the structured spatial component and the unstructured spatial component was assumed to be independently and identically distributed. Given a random vector $X=\left(x_{1},...,x_{n}\right)$the Besag model is defined as

$x_{i}/x_{j},i\neq j,\tau\sim N\left(\frac{1}{n_{i}}\sum_{i\neq j} x_{j},\frac{1}{n_{i}\tau}\right)$ (1)

where $n_{i}$the number of neighbours of region is$i$, $i\sim j$ indicates that two regions $i$ and $j$ are neighbours. The temporal random effect was assumed to be a random walk model of order 2 (RW2) constructed by assuming independent second order increments. Given a Gaussian vector $X=\left(x_{1},...,x_{n}\right)$ the RW2 model is defined as

$\Delta^{2}x_{i}=x_{i}-2x_{i+1}+x_{i+2}\sim N\left(0,\tau^{-1}\right)$ (2)

where $\tau$is the precision parameter.

Bayesian inference was carried out using the R library INLA 13] which implements the INLA method for latent Gaussian models to obtain small area estimates of malaria risk in the Kenyan coast.

In the GLMM the distribution of the response variable $Y_{i}$belongs to an exponential family of the form $Y_{i}/\theta_{i},\varphi_{1}\sim p\left(.\right)$where $p\left(\cdot\right)$ is a member of the exponential family defined as,

$p\left(y_{i}/\theta_{i},\varphi{}_{1}\right)=\exp\left(\frac{y_{i}\theta_{i}-b\left(\theta_{i}\right)}{a\left(\varphi_{1}\right)}+c\left(y_{i},\varphi_{1}\right)\right)$ (3)

for $i=1,...,n$ observations and $\theta_{1}$is the scalar canonical parameter. The mean $\mu_{i}=E\left(Y_{i}\right)$ can linked to the spatial and temporal random effects by a monotonic link function$g\left(\cdot\right)$ such that

$g\left(\mu_{\text{i}}\right)=\eta_{\text{i}}=\beta_{0}+\sum_{i=1}^{n} f_{i}\left(\mu_{\text{i}}\right)+\varepsilon_{\text{i}}$ (4)

where $f_{i}\left(\cdot\right)$ are random effects for model temporal and spatial dependencies, $\beta_{0}$ is the model intercept and $\varepsilon_{i}$is the residual error.

Latent Gaussian model a flexible and large class of statistical models obtained by assigning a Gaussian prior to$\beta_{0}$ ,$f_{i}\left(\cdot\right)$, and $\varepsilon_{\text{i}}$. This can be represented as $\Theta=\left(f_{i}'\text{s, }...\right)$ where $\Theta$ is unobserved multivariate Gaussian random variable, whose density $\pi\left(\Theta/\varphi\right)$ is controlled by a vector of hyperparameters $\Phi$^14^ . The latent Gaussian field $\Theta$is assumed to have a Gaussian distribution with zero mean and variance covariance matrix $Q\left(\varphi_{2}\right)$; with vector of hyperparameters defined as $\Phi=\left(\varphi_{1},\varphi_{2}\right)$ which are not necessarily Gaussian^13,15-16^.

Latent Gaussian model is composed of three elements namely; the likelihood of the data$\pi\left(y/\Theta\right)$, the Gaussian density of the random vector$\Theta$,$\pi\left(\Theta/\Phi\right)$ and the prior distribution of the parameter vector$\pi\left(\Phi\right)$. The posterior is therefore defined as

$\pi\left(\Theta,\Phi/y\right)\propto\pi\left(\Phi\right)\pi\left(\Theta/\Phi\right)\prod_{\text{i}=1} \pi\left(y_{i}/x_{i},\Phi\right)$ (5)

The main inferential interest involves computing the posterior marginals for $x_{i}$ and posterior marginals for$\Phi$ or some$\Phi_{j}$. Inference was implemented in Integrated Nested Laplace Approximations (INLA) developed by Rue and colleagues [13]. In INLA inferences are based on closed form approximations, which makes it to be computationally efficient^13^. The INLA methodology is described in the following section.

**INLA Methodology**

INLA is an approximate inference based method for approximating the posterior marginals of the latent Gaussian field $\pi\left(x_{i}/y\right),i=1....n$ in three steps^13^.

The posterior marginals of the latent effects $\Theta$and hyperparameters $\Phi$ are written as

$\pi\left(x_{\text{i}}/y\right)=\int\pi\left(x_{i}/\Phi,y\right)\pi\left(\Phi/y\right)d\Phi$ (6)

$\pi\left(\Phi_{\text{i}}/y\right)=\int\pi\left(\Phi/y\right)d\Phi_{-j}$ (7)

The posterior marginals $\tilde{\pi}\left(x_{\text{i}}/y\right)$ and $\tilde{\pi}\left(\Phi_{j}/y\right)$ can be approximated using the Laplace approximation. The first approximation to $\pi\left(\Phi/y\right)$using Gaussian distributions is constructed as follows

(8)

where $\tilde{\pi}_{G}\left(\Theta/\Phi, y\right)$ is a Gaussian approximation to the full conditional of $\Theta$ and $\Theta^{*}\left(\Phi\right)$ is the mode of the full conditional for $\Theta$, for a given value of $\Phi$. It involves locating the mode of $\tilde{\pi}\left(\Phi/y\right)$ which is used to integrate out the uncertainty with respect to $\Phi$ when approximating the posterior marginal of$x_{\text{i}}$.

The posterior marginals of the latent field are supposed to start from$\tilde{\pi}_{G}\left(x_{i}/\Phi,y\right)$ and approximate the density of $x_{i}/\Phi,y$with the Gaussian marginal derived from$\tilde{\pi}_{G}\left(\Theta/\Phi, y\right)$, i.e.

$\pi\left(x_{i}/\Phi,y\right)=N\left(x_{i};\mu_{i}\left(\Phi\right),\sigma_{ii}^{2}\left(\Phi\right)\right)$ (9)

The marginals of the interest can be computed using numerical integration over a multidimensional grid of values of $\Phi$

(10)

where the sum is over the values of $\Phi$ with area weights $\Delta_{k}$[Rue et al., 2007].

The first step in INLA computation involves approximating the posterior marginal of $\Phi$ by using Laplace approximation (8)

The second step involves computing the Laplace approximation of $\tilde{\pi}\left(x_{\text{i}}/\Phi,y\right)$ for selected values of $\Phi$ which improves the Gaussian approximation in equation (7).

(11)

where $\tilde{\pi}_{GG}\left(\Theta_{-i}/x_{i},\Phi,y\right)$ is a Gaussian approximation to $\Theta_{-\text{i}}/x_{i},\Phi,y$ around its mode $\Theta_{-i}\left(x_{i},\Phi\right)$. An improved version of $\tilde{\pi}_{LA}\left(x_{i}/\Phi,y\right)$ known as Simplified Laplace approximation was developed by Rue et al (2009). It involves a series of expansion of $\tilde{\pi}_{LA}\left(x_{i}/\Phi,y\right)$ around $x_{i}=\mu_{i}\left(\Phi\right)$ which corrects for skewness and location and it is also less computationally expensive^13^. The third step involves combining steps 1 and 2 using numerical integration in equation 10.

**Spatial Effects**

Suppose that the index $X=\left(x_{1},...,x_{n}\right)$ represents the geographically connected regions. The spatially correlated effects in INLA are introduced by assuming that neighbouring regions are more alike than two arbitrary regions. Two regions $x_{i}$ and $x_{j}$are neighbours if they share a common boundary and are assigned a value one and zero otherwise. The spatial smoothness prior for the function evaluation $f\left(x_{i}\right)$ is given by

$f(x_{i})/f(x_{j}),i\neq j^{'},\tau\sim N\left(\frac{1}{N_{i}}\sum_{i\sim j} f\left(x_{i}\right),\frac{1}{n_{i}\tau}\right)$ (12)

where $N_{i}$ are the number of neighbours of region$x_{i}$, $i\sim j$indicates that two regions $x_{i}$ and $x_{j}$are neighbours and $\tau$ is the precision parameter^17,18^. The model 12 assumes a conditional autoregressive (CAR) formulation and can also be referred to as Besag model. Assuming that the number found positive for malaria at location $i$at time $t$ is $Y_{it}$ out of the $N_{it}$ examined, then $Y_{it}$ is a binomial random variable, $Y_{it}\sim Bin(p_{it},N_{it})$, where $p_{it}$ is the proportion positive at each location $i$ at time $t$ . The logistic model incorporating both temporal and spatial effects as random effects is defined as

$\log\left(\frac{p_{it}}{1-p_{it}}\right)=f_{t}\left(time\right)+f_{\text{str}}\left(x_{it}\right)+f_{\text{unstr}}\left(x_{it}\right)$ (13)

where $f_{t}$ is time component, $f_{\text{str}}$and $f_{\text{unstr}}$are structured and unstructured spatial effects at sub location level. The $f_{\text{str}}$and $f_{\text{unstr}}$were assigned a Besag model prior and an independently and identically distributed Gaussian distribution respectively. The spatial effects were estimated at sub location level where their boundaries were used to compute the neighbourhood information. The temporal random effect was assumed to be random walk model of order 2 (RW2). That is, given a Gaussian vector $X=\left(x_{1},...,x_{n}\right)$ the RW2 model is defined as $\Delta^{2}x_{i}=x_{i}-2x_{i+1}+x_{i+2}\sim N\left(0,\tau^{-1}\right)$ where $\tau$is the precision parameter

Figure: The median *Pf*PR_2-10_ estimates by sub-location every five years from 1974-2014

**
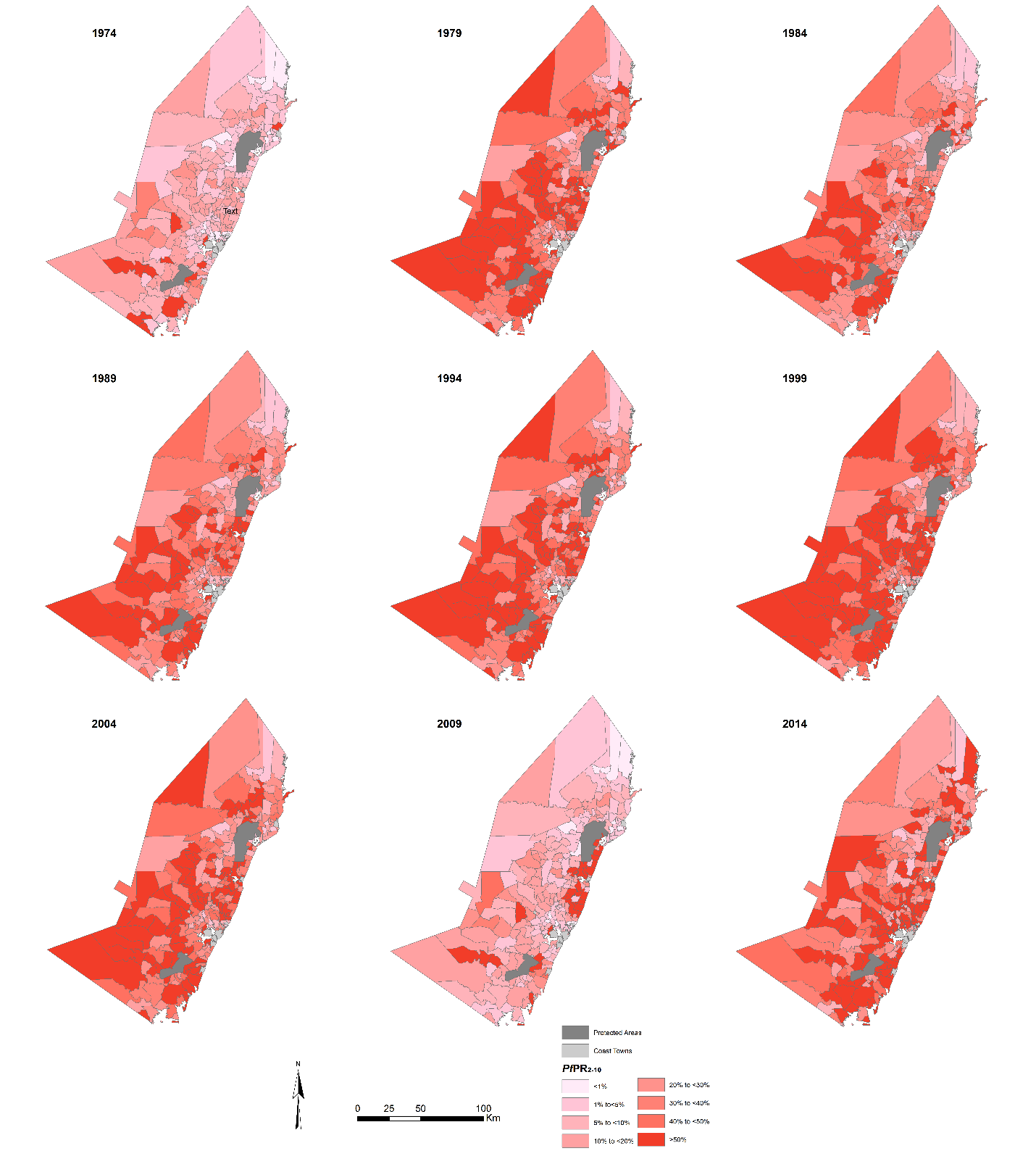
**

**References**

1. Gitonga CW, Karanja PN, Kihara J, Mwanje M, Juma E, Snow RW et al. Implementing school malaria surveys in Kenya: towards a nationwide surveillance system. [Malar J.](http://www.ncbi.nlm.nih.gov/pubmed/?term=Implementing+school+malaria+surveys+in+Kenya%3A+towards+a+nationwide+surveillance+system) 2010 Oct 30;9:306. doi: 10.1186/1475-2875-9-306.

2. Gitonga CW, Edwards T, **Karanja PN, Noor AM, Snow RW**, Brooker S (2012). Plasmodium infection, anaemia and mosquito net use among school children across different settings in Kenya. [Trop Med Int Health.](http://www.ncbi.nlm.nih.gov/pubmed/?term=Plasmodium+infection%2C+anaemia+and+mosquito+net+use+among+school+children+across+different+settings+in+Kenya) 2012 Jul;17(7):858-70. doi: 10.1111/j.

3. KEMRI-Wellcome Trust: http://www.kemri-wellcome.org/ (accessed 20^th^ January 2015)

4. KEMRI-Case-Western: http://www.case.edu/med/id/research_gh.html (accessed 20th January 2015)

5. Division of Malaria Control (DOMC). 2007 Kenya Malaria Indicator Survey. Division of Malaria Control Ministry of Public Health and Sanitation, March 2009

6. Division of Malaria Control [Ministry of Public Health and Sanitation], Kenya National Bureau of Statistics, and ICF Macro. 2010 Kenya Malaria Indicator Survey. Nairobi, Kenya: DOMC, KNBS, and ICF Macro, 2011

7. Gitonga CW, Kihara JH, Njenga SM, Awundo K, Noor AM, Snow RW et al. Use of rapid diagnostic tests in malaria school surveys in Kenya: does under-performance matter for planning malaria control? [Am J Trop Med Hyg.](http://www.ncbi.nlm.nih.gov/pubmed/?term=Use+of+rapid+diagnostic+tests+in+malaria+school+surveys+in+Kenya%3A+does+under-performance+matter+for+planning+malaria+control%3F) 2012 Dec;87(6):1004-11. doi: 10.4269/ajtmh.2012.12-0215

8. FIND. Foundation for innovative new diagnostics. New and improved technologies. Development and implementation of highly sensitive tools for endemic countries, September 2009 [cited September 7, 2011. http://www.finddiagnostics.org/programs/malaria/find_activities/new-improvedtechnologies/ (accessed 14^th^ January 2015)

9. Banerjee S, Carlin BP, Gelfand AE. Hierarchical Modeling and Analysis for Spatial Data. Chapman & Hall, New York, 2004

10. Best N, Richardson S, Thomson A. A comparison of Bayesian spatial models for disease mapping. [Stat Methods Med Res.](http://www.ncbi.nlm.nih.gov/pubmed/15690999) 2005 Feb;14(1):35-59

11. Singh BB, Shukla GK, Kundu D. Spatio-temporal models in small area estimation. Surv Methodol. 2005; 31: 183–196

12. Jackson C, Best N, Richardson S. Improving ecological inference using individual-level data. [Stat Med.](http://www.ncbi.nlm.nih.gov/pubmed/16217847) 2006 Jun 30;25(12):2136-59

13. Rue H, Martino S, Chopin N. Approximate Bayesian inference for latent Gaussian models by using integrated nested Laplace approximations. J R Stat Soc Series B Stat Methodol. 2009; 71: 319-92.

14. Rue H, Martino S. Approximate Bayesian inference for hierarchical Gaussian Markov random field models. J Stat Plan Inference. 2007; 137: 3177-92.

15. Martins TG, Simpson D, Lindgren F, Rue H. Bayesian computing with INLA: new features. Comput Stat Data Anal. 2013; 67: 68-83.

16. Fong Y, Rue H, Wakefield J (2009). Bayesian inference for generalized linear mixed models. [Biostatistics.](http://www.ncbi.nlm.nih.gov/pubmed/?term=Bayesian+inference+for+generalized+linear+mixed+models.+Biostatistics+11%3A+397-412.) 2010 Jul;11(3):397-412. doi: 10.1093/biostatistics/kxp053

17. Martino S, Rue H. Implementing approximate Bayesian inference using Integrated Nested Laplace Approximation: A manual for the INLA program. Department of Mathematical Sciences, NTNU, Norway, 2009

18. Brezger A, Kneib T, Lang S. BayesX: Analyzing Bayesian structured additive regression models. J Stat Spftw. 2005; 14: 1-22.
